# The contribution of individual psychological resilience in determining the professional quality of life of Australian nurses

**DOI:** 10.3389/fpsyg.2015.01613

**Published:** 2015-10-21

**Authors:** Desley G. Hegney, Clare S. Rees, Robert Eley, Rebecca Osseiran-Moisson, Karen Francis

**Affiliations:** ^1^School of Nursing and Midwifery, University of Southern QueenslandBrisbane, QLD, Australia; ^2^School of Psychology and Speech Pathology, Curtin UniversityPerth, WA, Australia; ^3^School of Medicine, The University of QueenslandBrisbane, QLD, Australia; ^4^Princess Alexandra HospitalBrisbane, QLD, Australia; ^5^School of Nursing, Midwifery and Paramedicine, Australian Catholic UniversityBallarat, VIC, Australia

**Keywords:** stress and coping, self-care, mental health, stress management, work/job satisfaction, resilience

## Abstract

**Research Topic:** The aim of this study was to determine the relative contribution of trait negative affect and individual psychological resilience in explaining the professional quality of life of nurses.

**Materials and Methods:** One thousand, seven hundred and forty-three Australian nurses from the public, private, and aged care sectors completed an online Qualtrics survey. The survey collected demographic data as well as measures of depression, anxiety and stress, trait negative affect, resilience, and professional quality of life.

**Results:** Significant positive relationships were observed between anxiety, depression and stress, trait negative affectivity, burnout, and secondary traumatic stress (compassion fatigue). Significant negative relationships were observed between each of the aforementioned variables and resilience and compassion satisfaction (CS). Results of mediated regression analysis indicated that resilience partially mediates the relationship between trait negative affect and CS.

**Conclusion:** Results confirm the importance of both trait negative affect and resilience in explaining positive aspects of professional quality of life. Importantly, resilience was confirmed as a key variable impacting levels of CS and thus a potentially important variable to target in interventions aimed at improving nurse’s professional quality of life.

## Introduction

Nurses work in stressful and demanding settings (Hegney et al., 2006; Eley et al., 2010) and often have less than optimal work conditions (such as long or inconsistent hours) which together can increase vulnerability to poor psychological functioning (Stamm, 2010). Indeed, a recent prospective study followed a cohort of 1,417 nurses in Sweden from point of graduation across the first 5 years of employment and found that one in every five nurses surveyed intended to leave the profession. Also, the authors found that the main predictor of intention to leave was level of burnout (Rudman et al., 2013). Internationally, studies have consistently reported high rates of burnout and other stress-related conditions among the nursing workforce (Ray et al., 2013; Hegney et al., 2014; Craigie et al., 2015). Conditions for which nurses may be particularly vulnerable to include stress-related conditions such as burnout, anxiety and depression, and secondary traumatic stress (STS; Figley, 1995; Stamm, 2010; Mealer et al., 2012). STS is one aspect of Compassion Fatigue (CF), a measure of the negative aspects of professional quality of life and refers to problems an individual may experience as a result of work-related trauma. This work-related trauma typically occurs in the form of secondary exposure as a result of working with patients who have had or are currently experiencing trauma. Such exposure can result in sleep difficulties, intrusive images, and avoidance of reminders of the traumatic experiences (Figley, 1995). CF is measured by the Professional Quality of Life Questionnaire (ProQol5; Stamm, 2010). The other component that makes up the overall construct of CF is burnout. Burnout in this context refers to emotional exhaustion, depersonalization, and reduction of personal accomplishments and is differentiated from CF as the former is related to general work-related stress whereas the latter is more specifically related to the practice of compassion (Stamm, 2010).

Whilst it is clear that the nursing workforce exhibit high levels of burnout and related negative psychological outcomes, some nurses demonstrate more positive psychological functioning. The positive psychology paradigm (see Seligman, 2002) aims to understand the factors that can explain why some individuals, despite being exposed to the same workplace stressors are able to maintain positive psychological functioning. Such efforts are important because if such factors are identified they can then be targeted in order to prevent deleterious outcomes in the workplace, such as burnout. Interventions that emerge from a positive psychology framework focus on building positive functionality as opposed to reducing pathology or negative symptoms. One construct that captures positive psychological functioning is Compassion Satisfaction (CS). In contrast to CF, CS refers to the positive aspects of professional quality of life. Specifically, CS is defined as the positive feelings one has about one’s professional work – the satisfaction a person receives through their work as a helper and when helping others (Stamm, 2010). An example of an item is ‘I get satisfaction from being able to help people.’ Studies have found higher CS to be associated with lower levels of burnout and STS among child protection workers (Conrad and Kellar-Guenther, 2006; Zerach, 2013) and lower burnout and depressed mood among nurses (Hegney et al., 2014). CS has also been found to act as a buffer against job strain (Tremblay and Messervey, 2011). Indeed, some authors have argued (e.g., Larsen and Stamm, 2008; Tremblay and Messervey, 2011), that individuals with high levels of CS may have more internal resources to buffer them against the effects of exposure to client trauma and occupational stress. Nurses who score highly on CS are more likely to be functioning well at work, have lower levels of burnout and STS (Stamm, 2010). Surprisingly less research attention has been directed toward CS as opposed to studies that have documented negative psychological outcomes among nurses (Ray et al., 2013).

Investigating the factors specifically related to positive psychological outcomes and the positive aspects of professional quality of life in nurses is necessary in order to determine which factors may be worthy of targeting in interventions. Resilience is one such factor that has received a great deal of research attention, initially in the 1980s and continuing today. Interest in the concept of resilience arose out of research which found that some individuals who were exposed to high level stressors were able to ‘recover, re-bound or adjust’ despite the adversity they had experienced (Garcia-Dia et al., 2013). The concept of resilience is acknowledged to be complex and multidimensional in nature (Waugh and Koster, 2014). The link between low levels of resilience and poor psychological functioning has been reported in numerous studies (Mak et al., 2011; Mealer et al., 2012; McGarry et al., 2013). Importantly, whilst it is generally agreed that resilience is partly a relatively stable and enduring characteristic, it has also been acknowledged that levels of resilience can and do change. In that sense it is not regarded as a static phenomenon, but rather a dynamic process, open to change and modification (Waugh and Koster, 2014).

**FIGURE 1:**
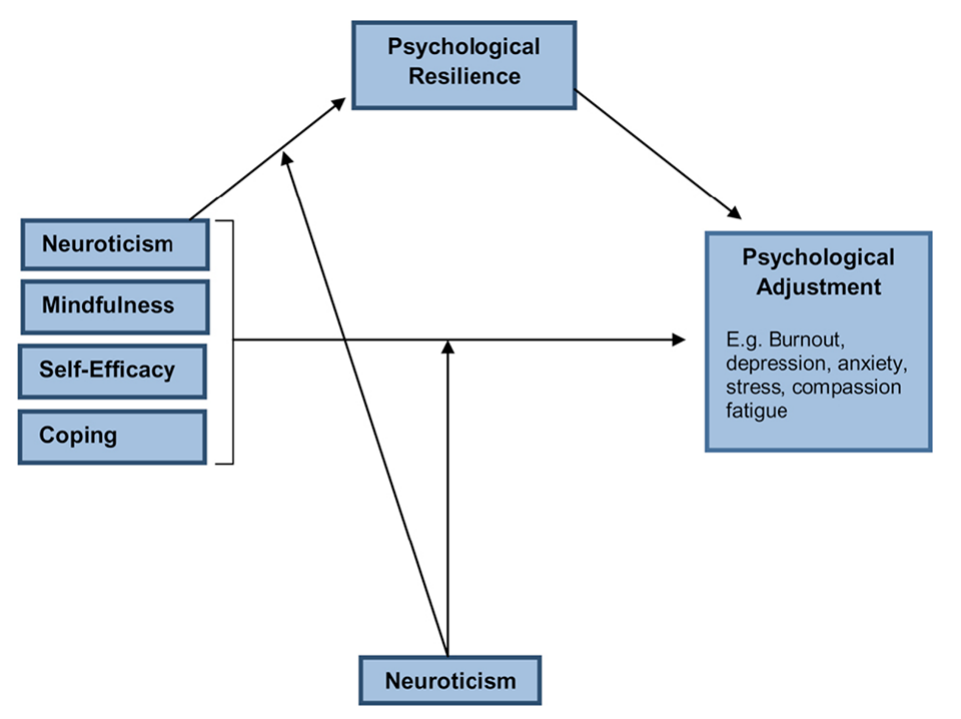
**ICWR1 Workforce Resilience Model (Rees et al., 2015)**.

Numerous studies have now reported high rates of stress-related conditions such as burnout, anxiety and depression, and STS among the nursing workforce as well as significant relationships among these variables. However, few studies have included a measure of resilience and examined the relationship between resilience and psychological outcomes. Furthermore, it is imperative that efforts are made to test the relative importance of resilience in explaining psychological functioning when examined alongside other key individual difference variables. With this in mind, Rees et al. (2015) recently put forward a theoretical model of individual resilience in the workplace that attempts to map essential key individual difference variables that together with resilience that may explain psychological functioning. In this model (see **Figure 1**) resilience is regarded as a critical individual difference factor that heavily influences the subsequent psychological functioning of a person. As can be seen in the model, a number of key variables are proposed as having a significant relationship with psychological functioning. These variables include Neuroticism, Mindfulness, Self-Efficacy, and Coping. Each of these variables has previously been shown to relate to levels of psychological functioning. For example, studies have shown that a high level of Neuroticism (also known as Trait Negative Affect) is consistently related to negative psychological outcomes such as high levels of depression and anxiety (Drury et al., 2014; Rees et al., 2014; Craigie et al., 2015). Similarly, low levels of mindfulness, self-efficacy, and adaptive coping behaviors have also been found to relate to negative psychological outcomes (Saks, 1994; Arch and Craske, 2010; Li and Nishikawa, 2012).

Mindfulness broadly refers to the ability of a person to effectively detach (de-center) from experiences, which facilitates a more balanced and less reactive response to those experiences. For example, a nurse who encounters a critical patient or staff member might ordinarily become distressed and upset by this interaction and may find it difficult to de-center from it, resulting in prolonged emotional distress that could then effect the entire working day. Alternatively, a nurse high in mindfulness would be able to de-center from the experience, appraise it in a more balanced way and although the experience may be unpleasant they would be more likely to be able to move on from it without the same level of ongoing distress. The construct of self-efficacy broadly refers to the belief a person has that he or she can effectively perform a given task. For example, a nurse who is low in self-efficacy may not believe that they have the ability to solve a problem at work. As such, low levels of self-efficacy have been found to relate to higher levels of stress and anxiety (Saks, 1994). Closely related to self-efficacy is coping. Coping strategies have been generally separated into those that are regarded as more helpful or adaptive and those considered less helpful/maladaptive. For example, planning and using humor are considered adaptive coping strategies whereas substance use and disengagement are considered maladaptive. The Rees et al. (2014) model proposes that each of the aforementioned variables (mindfulness, self-efficacy, coping) exert their influence on psychological functioning via the mediating role of resilience. The model identifies Neuroticism as an important and relatively stable individual factor that moderates the relationship between mindfulness, self-efficacy, coping, and psychological outcomes as well as between resilience and psychological outcomes (e.g., burnout, CS).

There are two aims of this study. The first is to build on the work of previous studies by examining correlations between resilience and other measures of psychological functioning among nurses. Second, to test one part of the recent theoretical model put forward by Rees et al. (2015); namely to determine if individual psychological resilience mediates the relationship between trait negative affect and psychological adjustment (CS). The following research questions were proposed:

(1) What is the relationship between individual psychological resilience and measures of psychological functioning in an Australian sample of nurses?
(2) Does individual psychological resilience mediate the relationship between trait negative affect and CS among Australian nurses?

## Materials and Methods

### Study Design

A cross-sectional survey design was employed in this study. As the main analysis consisted of multiple regressions, *a priori* power calculations were conducted. With four predictors a minimum of 74 participants was required to have sufficient power to detect a large effect according to Cohen’s conventions (Cohen, 1988).

### Participants

The inclusion criteria for the study was employment as a nurse or nursing assistant and membership of the Queensland Nursing Union in Australia. As the Union requested the research to be conducted it was a requirement that all eligible nurses within the Union be sampled. This meant, both nurses and nursing assistants were included in the overall sample. Nurses from three sectors were included if they were currently employed in the: private and public sectors (comprised of respondents from acute hospitals and community nursing sites) and aged care (comprised of public [government] and for-profit and not-for-profit [private] providers).

To achieve an equal representation across sectors, nurses who identified as being in the private (*n* = 4588) and aged care (*n* = 4661) sectors and who had valid email addresses were invited. Additionally 4500 public sector nurses were chosen randomly (using a random numbers table) from the more than 30,000 nurses in the public sector. Invitations were sent to 13,739 members. Of those, 2857 nurses began the Qualtrics survey of which 178 surveys were blank and thus excluded. This left 2679 (19.50%) nurses who had completed the whole or part of the survey. Of these nurses, a further 93 (3.47%) were excluded because they indicated that they were not employed, bringing the total number of responses to 2586 (response rate of 18.82%). For the purpose of this article only nurses who had completed all demographic questions plus the scales included in this study were retained which represented 1743 nurses (response rate 12.69%). To manage missing data we utilized the recommendations for each study measure and excluded cases if they exceeded the recommended number of missing items. Mean replacement was used for cases that did not exceed the missing item guidelines.

### Measures

*Demographics* (type of nurse [enrolled, registered, assistant in nursing]), age, sex, employment type [part-time or full-time], type of workplace of current employer [sector], nursing grade [for registered and enrolled nurses only], and length of time in nursing.

*Depression, Anxiety, and Stress Scale* (DASS; Lovibond and Lovibond, 2004) was used to measure mood symptoms over the past week. This study used the 21-item version of the longer 42 item scale. The DASS contains three sub-scales: depression, anxiety and stress. Each sub-scale consists of seven statements that are rated on a four-point Likert scale (0 – not at all, to 3 – very much/most of the time). Sample subscale items include: Depression – “I felt down-hearted and blue”; Anxiety – “I felt scared without any good reason”; Stress – “I tended to over-react to situations”. Each subscale is summed to obtain a full-scale score for the respective subscale. The DASS and DASS21 have demonstrated high internal consistency and strong psychometric properties in both normal and clinical populations (Brown et al., 1997; Antony et al., 1998; Lovibond and Lovibond, 2004). DASS21 scores are multiplied by a factor of 2 to obtain a full-scale score with an equivalent range to the DASS. Observed Cronbach’s alphas for each subscale were 0.89, 0.86, and 0.92 for stress, anxiety and depression, respectively.

*Spielberger State-Trait Anxiety Inventory form Y2* (STAI-Y2; Spielberger et al., 1983) was used to measure the construct of trait negative affectivity (TNA) – the tendency to experience a variety of negative emotions across a range of situations (Watson and Clark, 1984). Previous confirmatory factor analysis has confirmed that the STAI-Y2 is a valid measure of TNA (Bados et al., 2010). The STAI-Y2 consists of 20 items both positively and negatively worded that are measured on a four-point Likert scale (1 – almost never, to 4 – almost always). Higher scores indicate more trait negative affect. Mean scores reported in the general community are 36.35 (sd, 11.39) with the possible range of scores being 20–80 (Crawford et al., 2011). Respondents are asked to rate statements according to how the statements describe how they “generally feel”. Sample items include, “I feel nervous and restless,” “I feel secure,” “I feel inadequate,” “I feel like a failure,” “I get in a state of nervous tension when I think over my recent concerns and interests.” The measure is widely used in research and clinical settings, has sound psychometrics (Spielberger et al., 1983) and normative data in an Australian adult sample has been established (Crawford et al., 2011). The reliability for this study was α = 0.92.

*Professional Quality of Life Scale version 5* (ProQoL5; Stamm, 2010) utilizes 30 items that measure levels of CS and CF. The latter concept is composed of Burnout and STS. It uses a five-point item Likert scale (1 – never to 5 – very often) to measure the three subscales (ten items each). A score of 10 represents the lower score while 50 the higher score. Respondents are asked to read each statement in relation to their current work situation and select the number that reflects how “frequently they experienced these things in the last 30 days”. Sample items include: CS – “I feel invigorated after working with those I [help]”; BO – “I feel worn out because of my work as a [helper]”; STS – “I feel as though I am experiencing the trauma of someone I have [helped]”. The scale has been utilized internationally and also has been psychometrically validated in different studies for various target populations (Stamm, 2010). Observed Cronbach’s alphas for each subscale in this study were 0.91, 0.85, and 0.82 for CS, BO, and STS, respectively.

*Connor-Davidson Resilience Scale* (CD-RISC25; Connor and Davidson, 2003) was developed as a survey based measure of stress, coping, ability or resilience. Evidence from previous studies in the community (Hegney et al., 2008) and nursing populations (Gillespie, 2007) suggests that this scale is a valid and reliable measure of resilience (Connor et al., 2003). High internal consistency (*r* = 0.89), test-retest reliability (*r* = 0.87) and discriminant validity (0.83) has been reported (Connor et al., 2003). This 25 item scale uses a five point response scale. The total score ranges from 0 to 100. Reported mean scores have ranged from 68.0 (psychiatric outpatients) to 80.7 (US general community samples). Higher scores reflect greater resilience. The reliability for this study was α = 0.94.

### Procedure

The Human Research Ethics Committees of both Queensland University and Curtin University approved the study. Each of these ethics committees conforms to the national statement on ethical conduct in research involving humans. Emails to participate in the study were sent by the Queensland Nursing Union via their membership email database. The email contained a letter of invitation explaining the study, the Participant Information Sheet and the link to the on-line Qualtrics survey. Participants were informed that by beginning the survey they were providing their consent to take part in the study. The study was anonymous with no identifying data collected. The entire survey was estimated to take approximately 20–30 min to complete. A total of four reminders were sent to all participants over the 6 weeks that the study remained live on the website.

### Data Analyses

All statistical analyses were performed with IBM-Statistical Package for the Social Sciences (IBM-SPSS) for Windows version 21. Comparisons between categories were made using descriptive and inferential statistical tools appropriate to the scale of measurement (Chi-square, ANOVA, Welch tests). To examine relationships between variables bivariate correlations were performed. Two hierarchical regression analyses were used to test the possible mediating role of resilience.

## Results

### Demographics

The total sample of 1743 respondents were distributed fairly evenly between sectors: 27.08% (*n* = 472) of nurses worked in public setting, 31.44% (*n* = 548) in private and 28.74% (*n* = 501) in aged care. Furthermore there were 12.71% (*n* = 222) of nurses who were categorized in ‘other’ setting (e.g., practice nurses, nurses working in academic settings). The overall sample was predominately female (92.13%, *n* = 1604, *p* > 0.05). Participants were mainly (*p* < 0.05) registered nurses in public (86.99%, *n* = 408), private (78.79%, *n* = 431) and other (73.64%, *n* = 162) settings while the majority in aged care were nursing assistants. Nurses working in public (45.99, sd 11.12) and private sectors (46.53, sd 11.43) were a similar age (*p* > 0.05) and were younger than nurses working in aged care (49.43, sd 11.78) and in ‘other’ setting (50.26, sd 9.31). Nurses in aged care (72.85%, *n* = 365) tended to work more part time (*p* < 0.05) than nurses in the others settings. On average nurses in public, private and ‘other’ setting had been working in the nursing environment for 20 years while nurses in aged care had been working significantly less years on average (16.20, sd 14.26; *p* < 0.05). To clarify, in Australia permanent staff work either full or part time and have employment contracts whereas ‘casual staff’ do not have fixed contracts and as such do not have access to hospital benefits or hospital funded continuing professional education.

### Mean Scores and Standard Deviations for Study Measures

**Table 1** presents the means and standard deviations for each measure across each sector (*N* = 1743). Levels of depression (*M* = 5.46, *SD* = 7.76), anxiety (*M* = 4.38, *SD* = 6.39) and stress (*M* = 8.87, *SD* = 8.00) in the overall sample were higher than published Australian community norms (depression *M* = 2.57; anxiety *M* = 1.74; stress *M* = 3.99; Crawford et al., 2011). Significant differences were observed between the sectors for the Anxiety sub-scale of the DASS, where nurses in aged care reported significantly higher anxiety than those in the ‘other’ sector but there were no differences in anxiety scores between nurses in public, private or aged care sectors. The mean score on the Spielberger State-Trait Anxiety Inventory, measuring trait negative affect was 37.85 (*SD* = 9.93), which is comparable to published Australian community norms (*M* = 36.35, *SD* = 11.39; Crawford et al., 2011). The mean score for the total sample on the Connor-Davidson Resilience Scale was 70.02, somewhat lower than previously reported means from community samples. Overall, scores on the Professional Quality of Life Scale (CS *M* = 39.68, burnout *M* = 22.26, STS *M* = 20.29) were similar to other published means in nursing samples (Potter et al., 2010). CS scores were significantly higher for nurses working in the aged care sector compared to all other sectors and these nurses (aged care sector) also reported significantly higher STS than those in the public sector.

**Table 1:** Means and standard deviations for each measure across sectors.

	Public sector (*n* = 472)				Private sector (*n* = 548)				Aged care sector (*n* = 501)				Other sector (*n* = 222)				*P*-value^∗^
	Mean	*SD*	Min	Max	Mean	*SD*	Min	Max	Mean	*SD*	Min	Max	Mean	*SD*	Min	Max	
CD_RISC25	70.14	12.15	23.00	96.00	69.64	13.33	0.00	96.00	70.16	13.20	13.00	96.00	70.35	12.66	40.00	95.00	
TNA	37.83	9.33	20.00	74.00	37.98	10.07	20.00	69.00	38.09	10.16	20.00	72.00	37.04	10.31	20.00	79.00	
DASS Stress	8.57	7.54	0.00	38.00	9.35	8.29	0.00	42.00	8.93	8.04	0.00	42.00	8.20	8.08	0.00	40.00	
DASS Anxiety	3.94	5.63	0.00	36.00	4.69	7.13	0.00	42.00	4.82	6.45	0.00	40.00	3.57	5.75	0.00	42.00	Significance
DASS Depression	5.13	7.18	0.00	42.00	5.64	8.45	0.00	42.00	5.66	7.64	0.00	42.00	5.32	7.48	0.00	40.00	
ProQoL5 CS	38.78	6.43	11.00	50.00	39.21	6.89	14.00	50.00	41.13	6.06	14.00	50.00	39.53	6.33	18.00	50.00	Significance
ProQoL5 Burnout	22.65	5.81	10.00	42.00	22.29	6.24	10.00	47.00	22.06	6.27	10.00	48.00	21.85	5.76	11.00	43.00	
ProQoL5 STS	19.63	5.68	10.00	42.22	20.51	5.86	10.00	43.00	20.88	6.65	10.00	50.00	19.83	6.00	10.00	42.00	Significance

*CD-RISC25 refers to the 25-item of the Connor-Davidson Resilience Scale; STAI, Spielberger Trait Anxiety Inventory –Y2; TNA, Trait Negative Affect; DASS to the 21-item Depression, Anxiety, Stress Scale; ProQoL5, Professional Quality of Life version 5; CS, Compassion Satisfaction; Burnout; STS, Secondary Traumatic Stress.*

*^∗^P-value significant at <0.05.*

**Table 2** displays the correlation matrix for the variables age, years in nursing, DASS-21, CD-RISC25, STAI, and ProQoL5. As can be seen, age was significantly related to higher levels of CS and years in nursing was significantly and negatively correlated with the anxiety sub-scale of the DASS-21. Significant positive relationships were observed between anxiety, depression, and stress (DASS-21), trait negative affect (STAI), burnout and STS (ProQoL5). Significant negative relationships were observed between each of the aforementioned variables and resilience (CD-RISC25) and CS (ProQoL5).

**Table 2:** Bivariate correlations (Pearson) years in nursing, STAI-TNA, CD-RISC25, DASS, and PROQOL5 Subscales.

	Years in nursing	STAI-TRAIT	CD-RISC25	DASS			PROQOL5		
				Stress	Anxiety	Depression	CS	Burnout	STS
Age^a^	0.655^∗∗^	-0.182^∗∗^	0.120^∗∗^	-0.142^∗∗^	-0.176^∗∗^	-0.114^∗∗^	0.177^∗∗^	-0.174^∗∗^	-0.097^∗∗^
Years in nursing^b^		-0.147^∗∗^	0.107^∗∗^	-0.137^∗∗^	-0.181^∗∗^	-0.112^∗∗^	0.062^∗^	-0.128^∗∗^	-0.093^∗∗^
TNA			-0.680^∗∗^	0.703^∗∗^	0.607^∗∗^	0.707^∗∗^	-0.538^∗∗^	0.751^∗∗^	0.558^∗∗^
CD_RISC25				-0.466^∗∗^	-0.409^∗∗^	-0.514^∗∗^	0.628^∗∗^	-0.625^∗∗^	-0.354^∗∗^
DASS Stress					0.759^∗∗^	0.795^∗∗^	-0.351^∗∗^	0.633^∗∗^	0.573^∗∗^
DASS Anxiety						0.731^∗∗^	-0.292^∗∗^	0.518^∗∗^	0.543^∗∗^
DASS Depression							-0.430^∗∗^	0.647^∗∗^	0.495^∗∗^
ProQoL5 CS								-0.686^∗∗^	-0.231^∗∗^
ProQoL5 Burnout									0.607^∗∗^

*^a^n = 1714 (29 missing data), ^b^n = 1729 (14 missing data).*

*^∗^Significance at alpha level 0.05 (one-tailed); ^∗∗^significance at alpha level 0.01 (one-tailed). TNA refers to the Trait Negative Affect; DASS to the 21-item Depression, Anxiety, Stress Scale; CD-RISC25 to the 25-item Connor-Davidson Resilience Scale; STAI, Spielberger Trait Anxiety Inventory –Y2; ProQoL5 to the Professional Quality of Life version 5; CS, Compassion Satisfaction; Burnout.*

With respect to the ProQoL5 scale the nurses fell into the expected standardized ranges with CS having 24.7% in low category, 49.7% being average and 25.6% having high CS. Similarly, Burnout ranges were 29.0% in low category, 42.6% being average, and 25.6% having high burn out. Finally, STS ranges were 23.4% in low category, 54.4% being average and 22.2% having high STS.

**FIGURE 2:**
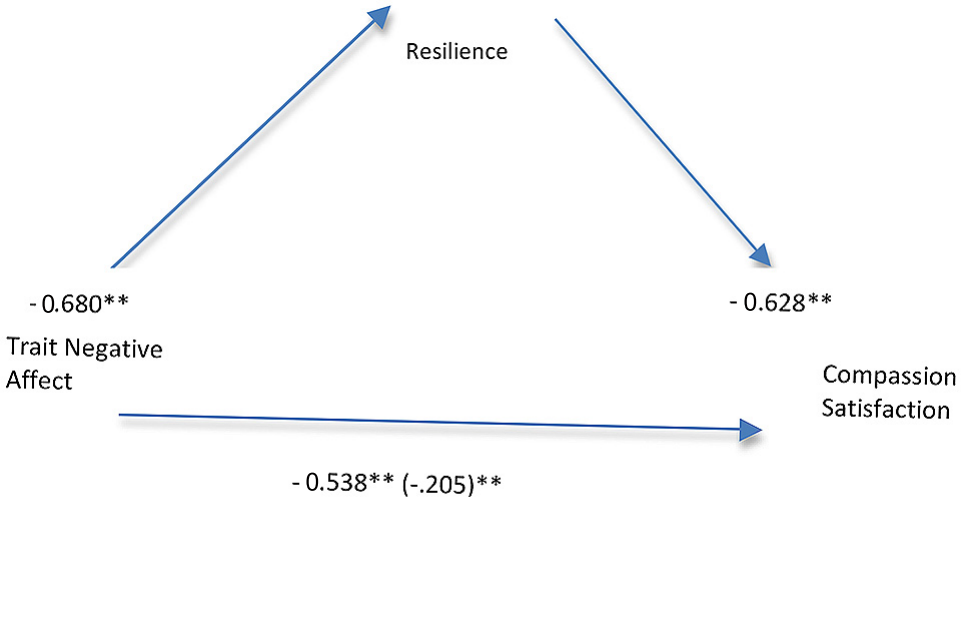
**Path analysis results testing model with resilience mediating the relationship between trait negative affect and compassion satisfaction.** All path coefficients shown are standardized. ^∗∗^*p* < 0.001.

One standard and one hierarchical multiple regression analysis was conducted to determine if resilience mediated the relationship between trait negative affect and CS. The results are presented in **Table 3** and as a diagram in **Figure 2**. The results from the standard hierarchical regression demonstrate that the IV (trait negative affect) accounted for 46% of the variance in the proposed mediator, resilience scores, *R*^2^ = 0.46, *F*(1,1741) = 1501.50, *p* < 0.001. On step one of the hierarchical multiple regression, resilience scores accounted for 39% of the variance in CS scores *R*^2^ = 0.39, *F*(1,1741) = 1135.72, *p* < 0.001. On step two of the hierarchical multiple regression trait negative affect scores were entered and accounted for a further 2.3% of the variance in symptom severity, Δ*R*^2^ = 0.023, *F*(1,1740) = 67.55, *p* < 0.001. In total, the two predictor variables accounted for 41.7% of the variance in CS scores. As can be seen in **Figure 2**, the pathway between resilience and CS remains significant after controlling for trait negative affect. Additionally, although the strength of the relationship between trait negative affect and CS is reduced when accounting for resilience, it remains significant. Taken together these results are consistent with partial mediation. Sobel test-statistics confirm that resilience significantly mediated the relationship between trait negative affect and CS (*z* = –17.02, *p* < 0.001).

**Table 3:** Results of Mediated Regression Analysis.

Standard regression (DV – Resilience)		*R*	*R^2^*	*ΔR^2^*	Beta
Step 1					
	Trait negative affect	0.680	0.463^∗∗^	0.463	-0.680^∗∗^

**Hierarchical regression (DV – Compassion satis.)**		***R***	***R^2^***	**Δ*R*^2^**	**Beta**

Step 1					
	Resilience (CD_RISC25)	0.628	0.395^∗∗^	0.395	0.489^∗∗^
Step 2					
	Trait negative affect (STAI)	0.646	0.417^∗∗^	0.023	-0.205^∗∗^

*^∗∗^p < 0.001.*

## Discussion

In this study, we sought to explore levels of psychological functioning and professional quality of life among Australian nurses. One of the primary aims was to test a key aspect of the recently proposed theoretical model of workplace resilience proposed by Rees et al. (2015) to determine the role that resilience plays in explaining a nurse’s level of CS, an index of psychological wellbeing. Specifically, Rees et al. (2015) propose that individual psychological resilience acts as a mediator between several other key psychological variables and subsequent psychological functioning. A major variable in the model is Neuroticism or trait negative affect and it is proposed that the relationship between this variable and psychological functioning is mediated by level of resilience. If true, this has important implications because it is generally agreed that it is possible to enhance levels of resilience, whereas trait negative affect is a more stable and enduring individual characteristic.

As proposed by Rees et al. (2015) both trait negative affect and individual psychological resilience explained significant variance in scores on CS. Importantly, resilience was confirmed to be a partial mediator of the relationship between trait negative affect and CS. In other words, whilst trait negative affect has a direct and significant relationship with CS, this relationship reduces in size from large (0.53) to relatively small (0.20) when resilience is added to the equation. This finding lends strong support to the notion that intervening to bolster nurse’s resilience is a worthy goal given that resilience ultimately influences professional quality of life, in this case, CS.

As expected, we found significant relationships between scores on the Professional Quality of Life Index (CS, STS, Burnout), depression, anxiety, stress, trait negative affect and resilience. Strong relationships (correlations between 0.5 and 0.7) were observed between trait negative affect and all other measures as well as between resilience and all other measures. The observed relationships between variables is consistent with previous findings (Hegney et al., 2014; Craigie et al., 2015). Levels of burnout and STS in our sample were 25.6 and 22%, respectively, a finding that is consistent with rates of CF reported previously by Hegney et al. (2014).

Whilst the current findings are encouraging, there are several limitations that must be taken into consideration when drawing conclusions. First, the response rate for the current study was low. This may have been due to the online and anonymous nature of the survey, making it easier for nurses to elect not to take part. Whilst the response rate in this study was low, this level of response does occur in online surveys, with rates as low as 9% being reported (Braithwaite et al., 2003). Also, the voluntary nature of the study means that the sample may be biased and not representative of the larger population of nurses. We did not exclude individuals with existing psychological disorders and as such the inclusion of such individuals may also have affected the generalisability of the results.

As a result of the modest response rate we temper our conclusions regarding the representativeness of the current findings, as we do not have information about the substantial portion of nurses who did not respond. Furthermore, due to the original purpose of this study, which was to sample a broad range of nurses from the Queensland nursing Union, our sample included nursing assistants. Nursing assistants have different roles to registered nurses and so may not be representative of the larger nursing workforce. However, the largest group in our study were registered nurses (68.47%) and thus the majority of the sample was more likely to be representative. Along similar lines, this sample was from only one state of Australia and so we cannot generalize to other nurses in other states. Despite that, the results here are consistent with rates of CF observed in a previous study with nurses from a different state in Australia (Hegney et al., 2014).

The findings of this study open up some important avenues for future research. As resilience is a complex construct, more research is required to further determine the key aspects of resilience, how best to measure it and ultimately how to enhance it. The present study has found support for the model proposed by Rees et al. (2015) confirming some important individual factors that are implicated in the overall emotional functioning of nurses. As this study only examined the mediating role of resilience using cross-sectional data, it will be important for future studies to test the model using data collected over several time-points. This will enable firmer conclusions as to which variables in the model best predict psychological functioning and whether resilience remains a mediator in this relationship. Whilst additional research is needed to further test individual factors we also suggest that more studies simultaneously consider the interaction between environmental and individual factors. We also believe that there should be studies that explore concepts of workplace resilience in the student nursing workforce as well as the employed nurse workforce.

## Conclusion

The results of this study support the importance of developing interventions that target resilience among nurses. The literature tells us that despite often stressful and challenging workplace situations, many nurses can maintain good levels of psychological functioning. This study provides evidence that resilience is an important variable in explaining CS and that this association is independent of the influence of stable personality factors such as trait negative affect. This finding offers much promise for the ability of targeted resilience interventions to build and promote emotional wellbeing among the nursing workforce. More work is required to determine what the critical elements of a targeted resilience-building intervention would be. Further testing of the Rees et al. (2015) model will assist with this task as the relationships between mindfulness, self-efficacy, coping, and individual psychological resilience remain to be fully tested.

## Conflict of Interest Statement

The authors declare that the research was conducted in the absence of any commercial or financial relationships that could be construed as a potential conflict of interest.
